# The efficacy and safety of multi-loop traction device for gastric endoscopic submucosal dissection: a single center prospective pilot study

**DOI:** 10.1038/s41598-023-47390-4

**Published:** 2023-11-16

**Authors:** Gen Kitahara, Toru Kaneko, Kenji Ishido, Yasuaki Furue, Takuya Wada, Akinori Watanabe, Satoshi Tanabe, Chika Kusano

**Affiliations:** 1 Department of Gastroenterology, Kitasato University School of Medicine, 1-15-1 Kitasato, Minami-ku, Sagamihara, Kanagawa 252-0373 Japan; 2 Department of Gastroenterology, Kitasato University Medical Center, Kitamoto, Japan

**Keywords:** Gastric cancer, Stomach

## Abstract

Although gastric endoscopic submucosal dissection (ESD) is widely used, the degree of difficulty varies greatly depending on the lesion. Since the multi-loop traction device (MLTD) has been suggested to shorten the procedure time in colorectal ESD, we examined the efficacy and safety of using the MLTD in gastric ESD. Thirty patients with gastric neoplasms were prospectively enrolled from February 2022 to December 2022, and the outcomes of ESD with the MLTD were evaluated. The primary outcomes were procedure time and dissection speed. The secondary outcomes were *en bloc* and R0 resection rates, MLTD attachment time, and complications of ESD with the MLTD. After excluding 1 patient, 29 patients (29 lesions) were treated by ESD with the MLTD. The median procedure time was 26 min (range, 9–210 min), and the median submucosal dissection speed was 39.9 mm^2^/min (12.4–102.7 mm^2^/min). The rate of *en bloc* resection was 100%, the median MLTD attachment time was 3 min (1–7 min), and none of the patients showed intraoperative or postoperative perforations. Thus, gastric ESD with the MLTD showed a favorable procedure time and dissection speed and an acceptable complication rate. Hence, the MLTD may be effective for gastric ESD.

## Introduction

Endoscopic submucosal dissection (ESD) allows *en bloc* resection of superficial tumors of the gastrointestinal tract and is minimally invasive in comparison with surgery. ESD also allows preservation of organ function after the procedure, thereby ensuring high postoperative quality of life in patients. In addition, the length of hospitalization for ESD is usually shorter than that for surgery, which can considerably improve healthcare economics^1^. The high rate of *en bloc* resection, which facilitates highly detailed histopathological evaluations and improves the curability of gastrointestinal neoplasms, has led to the widespread use of ESD, making it a routine clinical procedure^2–4^. Nevertheless, for gastric ESD, lesions located in the upper/middle body of the stomach, large lesions, or lesions with ulcer scars are considered key factors influencing the difficulty of the procedure and the risk of complications^5–7^.

During surgery, surgeons can use their non-dominant hand to generate counter-traction for the lesion and improve visualization of the operative field while resecting with their dominant hand. In contrast, endoscopists cannot use their non-dominant hand to generate traction, since the endoscope generally only has a single channel and, therefore, cannot generate effective counter-traction to dissect the submucosa^8^.

**Figure 1:**
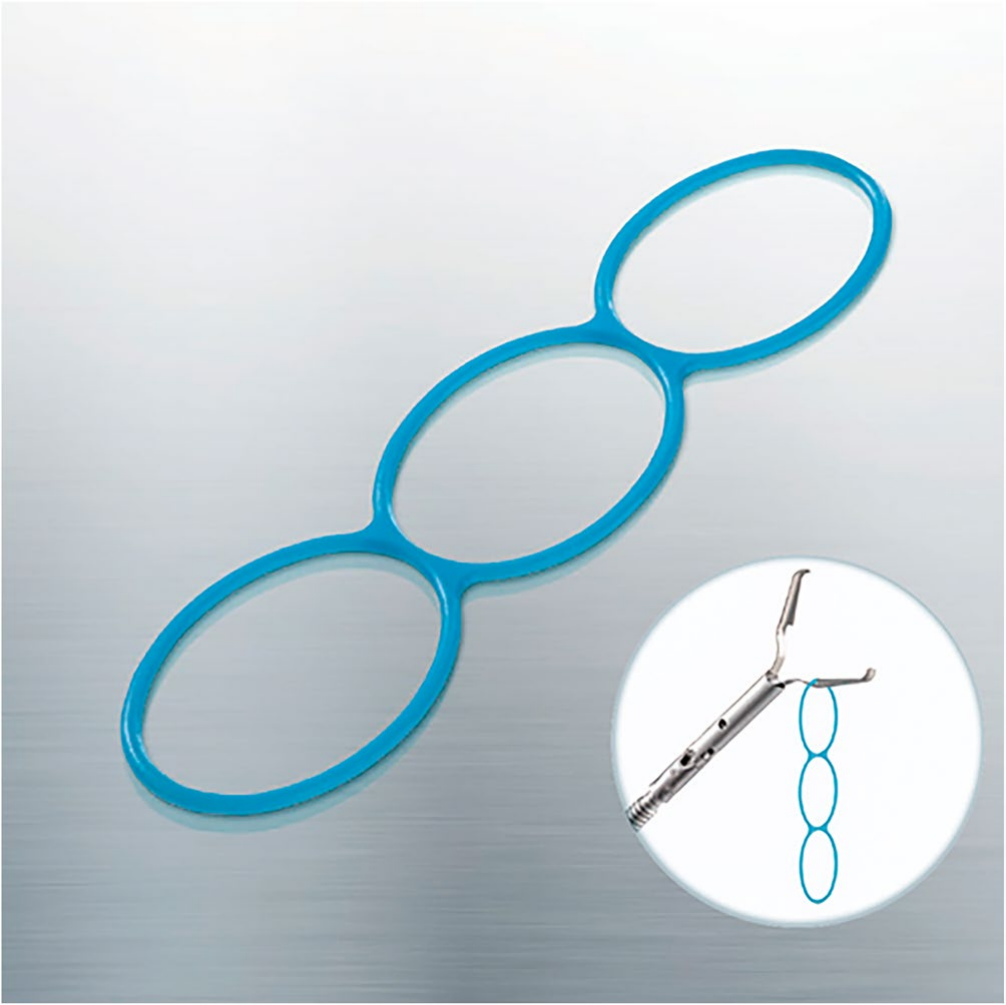
Multi-loop traction device (MLTD; Boston Scientific Co. Ltd., Tokyo, Japan).

Several traction techniques that can improve visualization of the submucosa, such as use of the clip-with-line^8,9^ and double-scope^10^ methods, have been reported to be effective for difficult lesions. In 2018, a Japanese multicenter randomized controlled trial showed that traction-assisted ESD using a clip with dental floss could reduce the risk of perforation and procedure time for appropriate lesions^11^. In 2021, a new traction device called the multi-loop traction device (MLTD; Boston Scientific Co. Ltd., Tokyo, Japan), which has three connected rings composed of unique linear biocompatible low-density polyethylene plastic, was released (Fig. 1). The MLTD can be delivered to the lesion easily from the channel of the endoscope and attached to the lesion and opposite side of stomach using a clip to generate counter-traction. In colorectal ESD, this new device was reported to be effective even in procedures performed by trainees^12^. In a previous ex vivo pilot study, MLTD was shown to increase the speed of submucosal dissection in gastric ESD and was similarly effective when used by expert and trainee endoscopists without causing perforations^13^.

Based on these findings, we hypothesized that the MLTD, a simple and convenient traction device, would be useful for gastric ESD. Therefore, this study aimed to evaluate the efficacy and safety of the MLTD for ESD of gastric neoplasms.

## Results

### Patient and tumor characteristics

**Figure 2:**
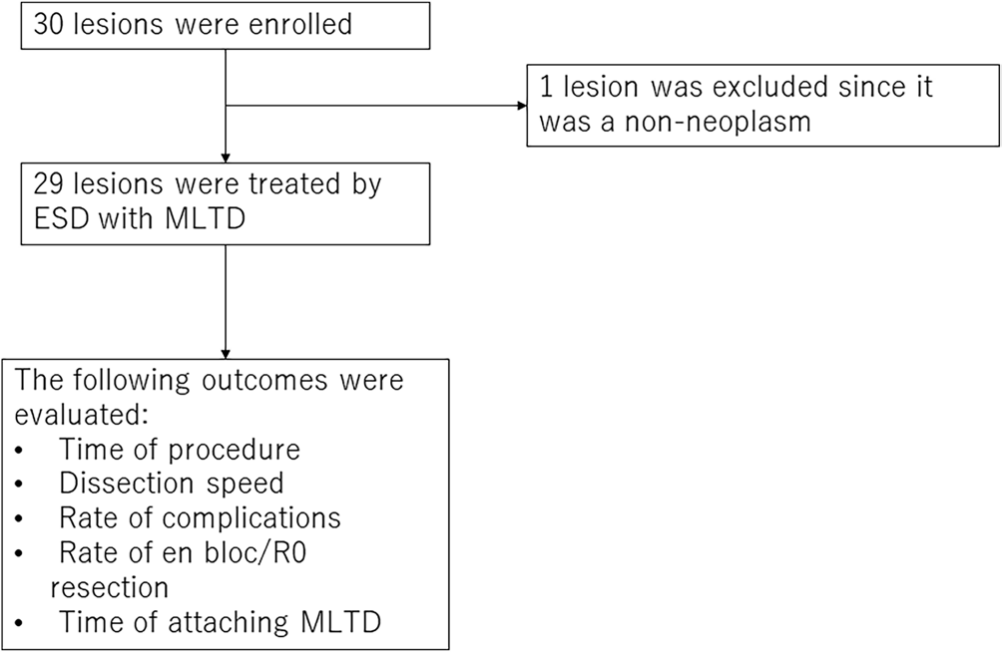
Patient enrollment protocol.

In total, 30 patients with 30 lesions were enrolled in this study; subsequently, 1 lesion was excluded because it was eventually diagnosed as a non-neoplasm (Fig. 2). The characteristics of the patients and tumors are shown in Table 1. The median age of the patients was 78 years (range, 62–88 years), and 20 patients were men (69%). Overall, 24 (83%) patients had good Eastern Cooperative Oncology Group performance status (PS 0–1), 22 (76%) were not receiving antithrombotic therapy, 5 (17%) were treated with a single drug, and 2 (7%) were treated with two or more drugs.

**Table 1:** Characteristics of the patients and tumors.

Characteristics	n = 29
Age: median (range), years	78 (62–88)
Sex: male/female	20/9
ECOG performance status: 0–1/2–	24/5
Charlson comorbidity index: 0–1/2–	13/16
Antithrombotic therapy: none/single drug/doublet or more	22/5/2
Tumor location: upper third/middle third/lower third	8/13/8
Macroscopic type: elevated/depressed/mixed	15/11/3
Atrophy: closed/open	5/24
Status of HP infection: current infection/previous infection/uninfected	5/23/1
Pathological diagnosis: adenoma/adenocarcinoma	3/26
Depth of tumor: M/SM1/SM2	26/1/2
Ulcer scar findings: yes/no	4/25
Histologic type: intestinal/diffuse	29/0

*ECOG* Eastern Cooperative Oncology Group, *HP Helicobacter pylori*, *M* mucosal invasion, *SM1* minute submucosal invasion (< 500 μm below the muscularis mucosae), *SM2* massive submucosal invasion (≥ 500 μm below the muscularis mucosae).

The lesion locations were as follows: upper third: U, 8 (28%); middle third: M, 13 (44%); and lower third: L, 8 (28%). Considering the macroscopic type, 15 (52%) lesions were of the elevated type (0-I or 0-IIa), 11 (38%) were of the flat/depressed type (0-IIb or 0-IIc), and 3 (10%) were of the mixed type (0-IIa + IIc). Overall, we diagnosed 3 lesions as adenomas (10%) and 26 as adenocarcinomas (90%). Four lesions appeared as ulcerative scars in the submucosa (14%).

### Outcomes

The outcomes of ESD with the MLTD are shown in Table 2. The median length and size of the specimens were 38 mm (range, 20–75 mm) and 942 mm^2^ (range, 282.6–2826 mm^2^), respectively. The median procedure time was 26 min (range, 9–210 min), and the median submucosal dissection speed was 39.9 mm^2^/min (range, 12.4–102.7 mm^2^/min). The rate of *en bloc* resection was 100%, and no intraoperative or postoperative perforations were observed. One patient who received doublet antithrombotic therapy showed delayed bleeding after the procedure.

**Table 2:** Outcomes of ESD with the MLTD.

Outcomes of ESD	n = 29
Procedure time, median (range), min	26 (9–210)
Procedure time without attachment time of the MLTD, median (range), min	22 (6–200)
Length of the specimen, median (range), mm	38 (20–75)
Size of the specimen, median (range), mm^2^	942 (282.6–2826.0)
Dissection speed, median (range), mm^2^/min	39.9 (12.4–102.7)
*En bloc* resection: yes/no	29/0
R0 resection: yes/no	29/0
Intraoperative bleeding: yes/no	0/29
Intraoperative perforation: yes/no	0/29
Delayed bleeding: yes/no	1/28
Delayed perforation: yes/no	0/29

*ESD* endoscopic submucosal dissection, *MLTD* multi-loop traction device.

The MLTD-related items are summarized in Table 3. The median MLTD attachment time was 3 min (range, 1–7 min). A total of 32 MLTDs were used. A single MLTD was used in 26 lesions, and two MLTDs were used in 3 lesions. The mean number of MLTDs used was 1.10. The MLTD was dislodged in three cases: reattachment of the MLTD was required in two of them, whereas the procedure was completed without reattachment in the third case. In one case, an additional MLTD was attached during the procedure to generate traction in a different direction. In another case, the traction direction was changed by attaching an unused ring of the MLTD to another point of stomach. Traction-related damage to the specimen was not observed in this study.

**Table 3:** MLTD-related items assessed.

MLTD-related items	
MLTD attachment time, median (range), min	3 (1–7)
MLTDs used per case: (1 MLTD/2 MLTDs)	26/3
Reattachment of MLTD due to dislodgement	2
Additional MLTD attached during the procedure	1
MLTDs dislodged: yes/no	3/29
Use of MLTD to change traction direction: yes/no	1/31
Traction-related damage to the specimen: yes/no	0/32

*MLTD* multi-loop traction device.

## Discussion

To the best of our knowledge, this is the first report about the efficacy and safety of MLTD for gastric ESD. The procedure time, dissection speed, and safety of this technique were favorable in this study.

Although several traction methods have been reported to facilitate ESD^10,14,15^, they were complicated, time-consuming, and have not been widely used for gastric ESD. The most commonly used traction technique involves a clip-with-line device and was assessed in CONNECT-G, a large prospective randomized controlled trial conducted in 2018^11^. CONNECT-G reported that the median procedure time of clip-with-line group was 58.1 min^11^ and concluded that this technique improved the procedure time and reduced the risk of perforation in cases showing lesions in the greater curvature of the upper or middle stomach, but it did not significantly reduce the overall procedure time. When using the clip-with-line traction method, the direction of traction is limited to a single direction on the cardiac side, which may not be effective depending on the location and size of the tumor. Therefore, the CONNECT-G trial did not show significant overall differences.

Gastric ESD procedures that use the S–O clip, which allow traction to be applied in any direction, have been reported^16,17^. The median procedure time of these reports was 45 min^16^ and 29.1 min^17^, and median dissection speed shown by Nagata M was 21.8 mm^2^/min^17^. Although these reports involved few cases, both reports concluded that the procedures were significantly time-efficient for cases located at U/M lesions of the stomach. In procedures performed using the S–O clip, the operator can choose the direction of traction. However, the procedures have the following disadvantages: the application procedure is somewhat complicated, the S–O clip is difficult to use in areas with strong flexion or a narrow lumen, and its traction is relatively weak as dissection progresses in large lesions.

Although this study did not directly compare the usefulness of the MLTD in gastric ESD with that of other traction devices, the ESD procedure time in this study was shorter than that reported previously (26 min vs. 29–58.1 min)^11,16,17^. Similarly, the dissection speed in this study was better than that reported previously (39.9 mm^2^/min vs. 21.8 mm^2^/min)^17^. In conventional ESD, for lesions with a field of view that was difficult to visualize, a situation-specific strategy was essential to dissect the area quickly and safely. However, in this study, ESD was performed using a simple strategy of making a circumferential incision first, followed by traction and dissection with the MLTD. In addition, the MLTD can be used effectively for all types of lesions because the traction force can be changed depending on the selected ring, even in areas with strong flexion or a narrow lumen. The previous reports with S–O clip show that the median attachment time was 4.4 min^16^ and 1.82 min^17^. In this study, the median MLTD attachment time was 3 min and considered to be suitable. Furthermore, the traction direction and force can be easily adjusted by changing the ring to be grasped during the procedure. Moreover, because of its simple application method and short application time, this technique could yield an improved submucosal field of view with an additional attachment. These factors may have contributed to the short procedure time and rapid dissection. The safety of this technique was comparable to that achieved using existing devices, and none of the patients showed bleeding or perforation^11^.

In CONNECT-G, 10 lesions (3.1%) in the traction group were damaged by the clip-with-line^11^. However, in the present study, no specimen damage was observed, although some cases showed dislodgement. The MLTD is weaker and easier to break than the thread; thus, when strong tension is applied to the lesion, the MLTD breaks before the specimen incurred damage.

This study had a few limitations. First, this was a prospective exploratory pilot study with a small sample size and no control group. Second, all procedures were performed at a single institution and by a single endoscopist, limiting the generalizability of the results. Thus, it was still unclear whether this new traction device was truly effective for gastric ESD. A multicenter randomized controlled trial with a large sample size comparing conventional traction method and MLTD focused on physician’s skill, tumor location, and traction devices was warranted to address these limitations.

In conclusion, gastric ESD with an MLTD showed a favorable procedural time and dissection speed along with an acceptable complication rate. Thus, the use of an MLTD may be effective for gastric ESD.

## Methods

### Study design and patient eligibility

This single-center prospective pilot study was conducted in accordance with the principles of the Declaration of Helsinki. The study protocol was approved by the institutional review board of Kitasato University Medical Center (protocol approval number: 2021029). All patients provided written informed consent prior to enrollment.

The number of gastric ESD cases at Kitasato University Medical Center was 40–50 per year. Although there was no statistical basis for designing this prospective study, 30 cases that could be accumulated in approximately one year were selected for the pilot study.

Consecutive patients who underwent ESD for gastric neoplasms at Kitasato University Medical Center between February 2022 and December 2022 were enrolled in this study (Fig. 2). The procedures were performed by a single endoscopist whose experience amounted to 150 procedures. Eligible patients were aged ≥ 20 years and were confirmed to have lesions that met the absolute or expanded indications of the Japanese Gastric Cancer Treatment Guidelines (version 6) for preoperative endoscopic examination^18^.

The exclusion criteria were as follows: history of gastrectomy, inability to cease anticoagulant or antiplatelet medications except for low-dose aspirin, active infection, pregnancy or breastfeeding, severe mental disorder, steroid dependence, myocardial infarction within 6 months or unstable angina pectoris within 3 weeks of the indications for ESD, severe respiratory disease requiring continuous oxygen therapy, unstable hypertension, and uncontrolled diabetes mellitus or insulin therapy.

### Statistical analysis

Categorical variables were expressed as number (%), whereas continuous variables with a non-normal distribution were expressed as median. All calculations were performed with the use of the Statistical Packages for the Social Sciences (SPSS), version 27 (IBM Corp.; Armonk, NY, USA).

### ESD procedure

Currently, various ESD devices are used, and dissection methods and strategies vary among institutions. In this study, all procedures were performed using the endoscopes GIF-Q260J or GIF-2TQ260M (Olympus, Tokyo, Japan), and IT Knife-2 (KD-611L; Olympus, Tokyo, Japan) was used as the dissection device.

**Figure 3:**
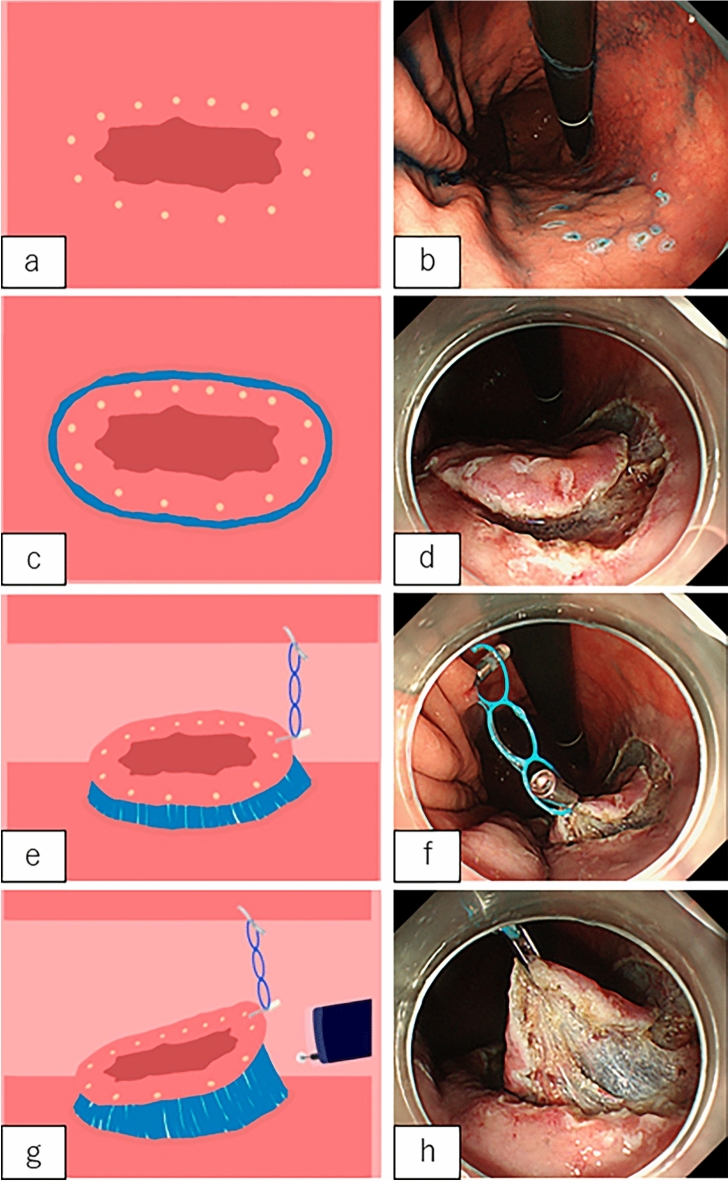
Endoscopic submucosal dissection (ESD) procedure using IT Knife-2 and the multi-loop traction device (MLTD). (**a**, **b**) Marking around the lesion (**a**: illustration; **b**: photograph). (**c**, **d**) Circumferential incision (**c**: illustration; **d**: photograph). (**e**, **f**) Attachment of the MLTD to the opposite side of the stomach (**e**: illustration; **f**: photograph). (**g**, **h**) Adequate traction to the lesion (**g**: illustration; **h**: photograph).

**Figure 4:**
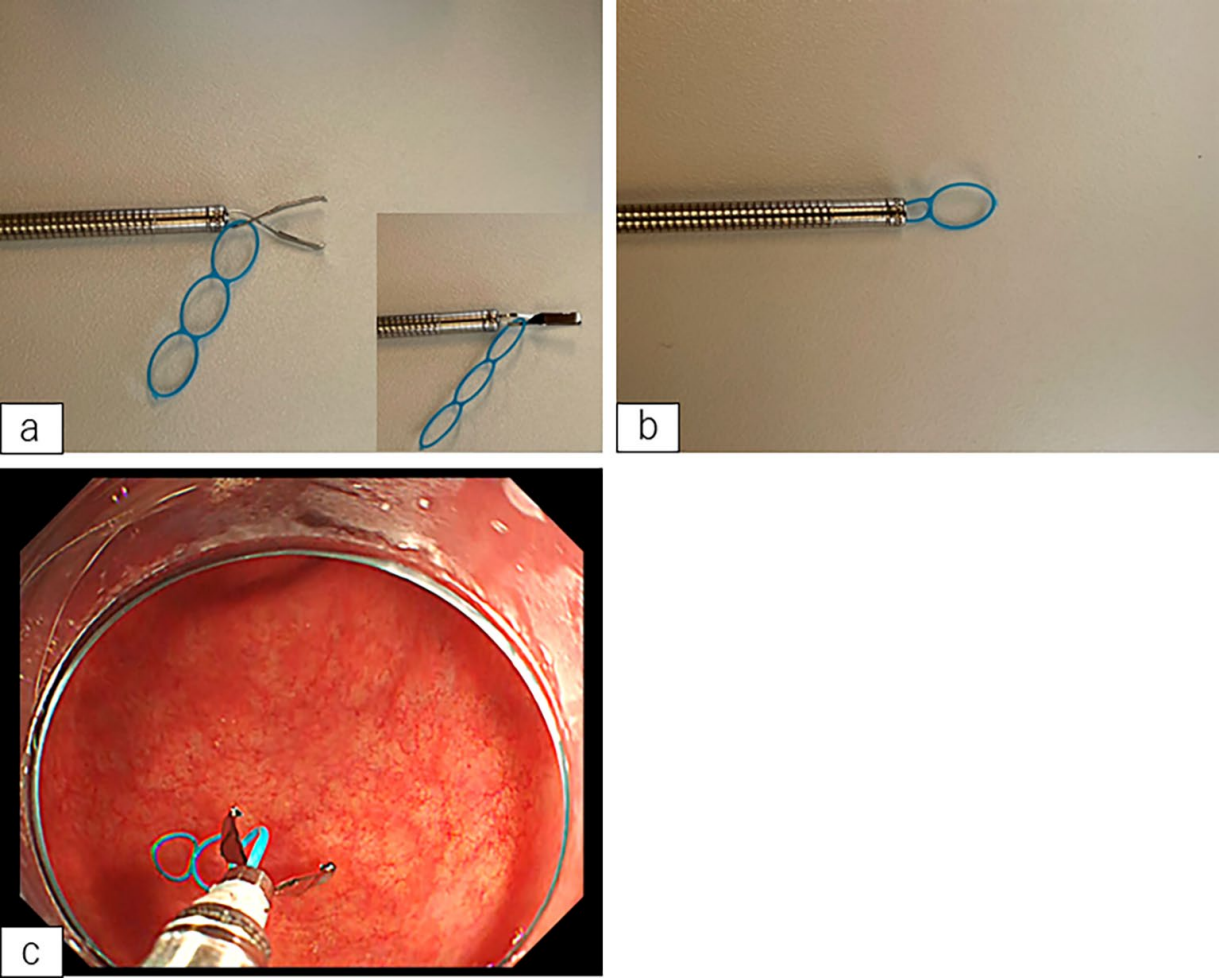
Images of attachment of multi-loop traction device (MLTD) to EZ clip. (**a**) Half-opened clip and MLTD at the base of the clip. (**b**) Clip with MLTD loaded to the catheter. (**c**) Endoscopic photograph of opening clip with MLTD.

The dissection method used is as follows: Circumference marking points outside the lesion were first created using a needle device (Fig. 3a,b). After injecting a mixture of normal saline and hyaluronic acid into the submucosa, an initial cut outside the mark was made using a needle device to insert the tip of IT Knife-2; this was followed by circumferential mucosal cutting (Fig. 3c,d). Subsequently, the MLTD was delivered to the lesion using an open/closed clip (EZ clip; Olympus or Sureclip; MCmedical) from the scope channel. When MLTD was used for EZ clip, the following procedure was performed. First, the clip was attached to the catheter as usual, after which, the clip was once half-opened and the MLTD was equipped at the base of the clip, and finally the clip with MLTD was loaded to the catheter and inserted through the channel of the endoscope (Fig. 4a–c).

**Figure 5:**
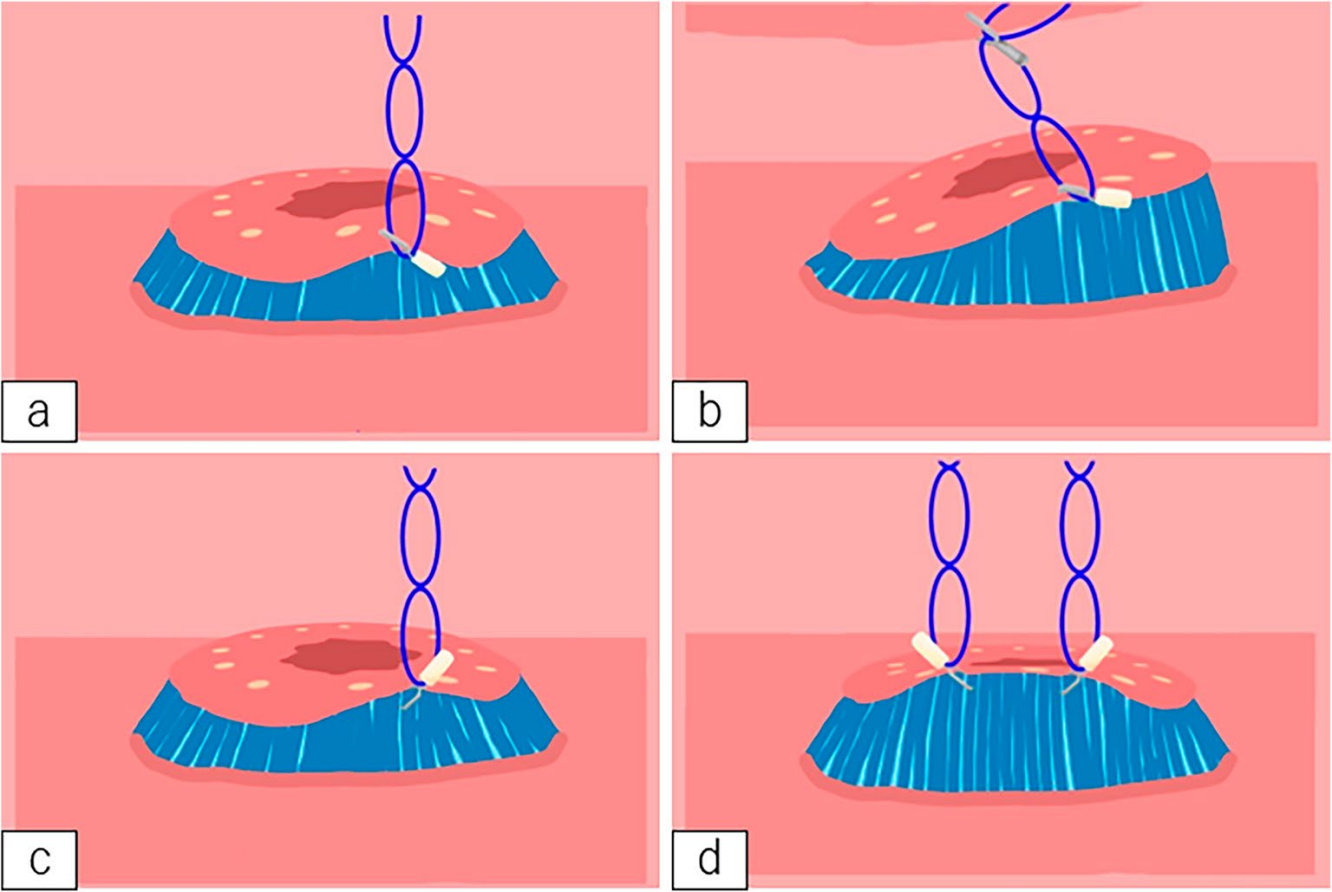
Illustrations of additional procedures performed with the multi-loop traction device (MLTD). (**a**, **b**) Changing the traction direction by grasping another ring. (**c**, **d**) Additional attachment of the MLTD.

The side ring of the MLTD was clipped to the edge of the lesion, and another ring was clipped to the opposite side of the stomach (Fig. 3e,f). The MLTD connecting the edge of the lesion and the stomach generated counter-traction to raise the lesion and improve visualization of the submucosa (Fig. 3g,h). Following re-injection into the submucosa, the submucosal dissection was continued using IT Knife-2. When the traction direction had to be changed during the procedure, another ring was grasped and clipped onto other parts of the stomach (Fig. 5a,b). When necessary, another MLTD was added to create additional traction and expand the submucosal field of view (Fig. 5c,d). After completing the procedure, the MLTD was torn off using grasping forceps.

### Histopathological evaluation

The tissue specimens were fixed in formalin, cut into 2-mm-wide strips perpendicular to the lesion base, and embedded in paraffin. A pathologist examined the sections for histopathological diagnosis according to the Japanese Classification of Gastric Carcinoma and the World Health Organization classification^18^. The tumor size, depth of invasion, presence of ulcerative changes, lymphatic and vascular involvement, and tumor involvement in the lateral and vertical margins were assessed. The depth of invasion was defined as follows: M, mucosal invasion; SM1, minute submucosal invasion (< 500 µm below the muscularis mucosae); and SM2, massive submucosal invasion (“≥ 500 μm below the muscularis mucosae).

### Antithrombotic therapy

For patients receiving antithrombotic therapy, drug withdrawal was performed in accordance with the Guidelines for the Management of Anticoagulant and Antiplatelet Therapy for Endoscopic Procedures issued by the Japan Gastroenterological Endoscopy Society in 2017^19^.

### Definitions

The procedure time was defined as the time from the first injection before initial cutting to the end of tumor dissection. The specimen area was calculated as follows: specimen area [mm^2^] = (transverse diameter [mm]/2) × (longitudinal diameter [mm]/2) × 3.14 (π). The dissection speed was calculated by dividing the specimen area by the procedure time without the MLTD attachment time.

*En bloc* resection was defined as endoscopic resection of an entire lesion in a single specimen. R0 resection was defined as endoscopic resection of an entire lesion in a single specimen with no histopathological evidence of tumor residue at the resection margins. Cases in which the margins of the lesion were unclear because of electrosurgical or mechanical damage were classified as non-R0 resections.

Perforation was diagnosed if intra-abdominal organs or fat tissues were observed during the procedure or free air was found on post-procedure radiographs or computed tomography images.

Delayed bleeding was defined as clinical evidence of bleeding after ESD, which required blood transfusion or urgent endoscopic intervention and/or a decrease of > 2 g/dL in the hemoglobin level on blood examination.

### Outcomes

The procedure time and dissection speed for gastric ESD with MLTD were recorded as the primary outcomes of this study. The *en bloc* and R0 resection rates, MLTD attachment time, and rate of complications during the procedure were investigated as secondary outcomes.

## Data Availability

The datasets used and/or analyzed during the current study available from the corresponding author on reasonable request.
